# The relationship between changes in inflammation and locomotor function in sensory phenotypes of central neuropathic pain after spinal cord injury

**DOI:** 10.1097/PR9.0000000000001184

**Published:** 2024-10-10

**Authors:** Brittany L. Avonts, Quan Shen, Neal J. Wrobel, Richard G. Fessler, Brian T. David

**Affiliations:** Rush University Medical Center, Department of Neurosurgery, Chicago, IL, USA

**Keywords:** Spinal cord injury, Central neuropathic pain, Inflammation, Hyposensitivity, Hypersensitivity

## Abstract

**Introduction:**

Central neuropathic pain (CNP) commonly develops in patients after spinal cord injury (SCI), causing debilitating symptoms and sensory abnormalities to mechanical and thermal stimuli. The biological variability of pain phenotypes in individuals has limited the number of positive outcomes. Thus, it is necessary to investigate the physiological processes contributing to sensory changes that develop over time.

**Objective:**

To investigate the physiological processes contributing to neuropathic pain sensory changes and locomotor impairments with sensory phenotypes that develop over time.

**Methods:**

Using the tail flick and von Frey tests, we performed hierarchical clustering to determine the subpopulation of rats that developed thermal and mechanical sensory abnormalities. To measure inflammation as a potential mediator of CNP phenotypes, we used flow cytometry and immunohistochemistry. Finally, to assess the secondary effects on locomotor recovery, up to 8 weeks after injury, we used the CatWalk test to assess multiple parameters of gait.

**Results:**

The von Frey test showed a subpopulation of SCI rats that were hyposensitive to mechanical stimuli from 6 to 8 weeks after injury. The tail flick test showed a subpopulation of SCI rats that were hypersensitive to thermal stimuli at 1 week and 3 to 8 weeks after injury. Although there were no differences in inflammatory cells between subpopulations, we did see significant changes in locomotor recovery between rats with and without sensory abnormalities.

**Conclusion:**

The myeloid cell population at large is not affected by mechanical or thermal phenotypes of pain in this model; however, locomotor recovery is impaired depending on the pain phenotype present. Further investigation into acute inflammatory cells may be insightful for predicting the development of pain phenotypes.

## Abstract 2

Supplemental Digital Content is Available in the Text.

Inflammation is not affected by mechanical or thermal phenotypes of pain after spinal cord injury. However, locomotor recovery is impaired dependent on the pain phenotype.

## 1. Introduction

Central neuropathic pain (CNP) develops in over half of individuals with spinal cord injury (SCI), greatly reducing one's quality of life through debilitating pain.^2,10,41,46^ Constant burning or stinging sensations commonly develop below the level of injury because of altered sensitivity. Central neuropathic pain is refractory to most therapies because of an incomplete understanding of its underlying mechanism and presentation.^3,11^ Comprehending the relationship between sensory and motor recovery, physiological processes, and the pathological mechanism of CNP development could yield insight into predictive biomarkers, optimal treatment timing, and therapeutic potential.

When SCI results in the development of CNP, the population of neurons responsible for distinguishing painful nociceptive stimuli from innocuous ones becomes disrupted, giving rise to sensory abnormalities.^44^ Sensitivity to mechanical and thermal stimuli are regularly measured to assess CNP in both animals and humans alike.^22^ In rodents, sensitivity to mechanical stimuli closely emerges with motor recovery a few weeks after a contusive injury.^25^ If we can better understand the presentation of sensory deficits and underlying pathological and behavioral consequences, we can provide insight to the complex mechanisms and find the most effective treatment strategy.

Inflammation within the spinal cord, regardless of the level, can alter the normal processing of sensory information because of the complex and dynamic nature of the spinal cord. Several studies have reported a T9/T10 contusion in rats resulted in activation of microglia in the cervical and low thoracic segments of the spinal cord.^1,38,47^ Although the lumbar and sacral spinal cord primarily process sensory information related to thermal and mechanical stimuli, injury to the thoracic region can exert distant effects which is crucial for understanding the comprehensive pathophysiology of the condition.

In the present study, we aimed to assess the role of inflammatory cells at the spinal cord injury epicenter on neuropathic pain using tests for both mechanical and thermal sensitivity. Unlike other studies which primarily use tests of evoked stimuli that rely on hind paw placement to measure pain sensitivity, we included an acute measure of thermal sensitivity to investigate the earliest timepoint at which abnormal evoked responses develop. We also examined how different sensory responses affect SCI locomotor recovery because pain can be known to influence functional recovery.^28^

## 2. Methods

### 2.1. Animals

Adult female Sprague Dawley rats (180–210 g; Envigo, Indianapolis, IN) were used in this study. Animals were kept in pairs with access to food and water ad libitum throughout the study, in a room with controlled temperature and humidity, under a 12-hour light–dark cycle. In total, 38 rats were included in the experiment, 11 rats were randomly assigned to receive a sham surgery and 27 rats to receive a SCI surgery. Force/displacement curves were inspected at the time of injury and the Basso-Beattie-Bresnahan locomotor rating scale (BBB) was administered at 1 dpi to confirm consistency of the injury; as a result 3 rats were excluded from this study.^5^

### 2.2. Surgery for spinal cord injury

All protocols and procedures were approved by the Institutional Animal Care and Use Committee (IACUC) of Rush University. Rats were deeply anesthetized and maintained on 3% isoflurane delivered through a nose cone. A laminectomy at thoracic vertebrae 9 (T9) was performed and a 200-kdyn, 0 dwell time spinal cord contusion was induced using the Infinite Horizon Impactor (Precision Systems and Instrumentation, Lexington, KY) as previously described.^17,18^ Sham rats only received a T9 laminectomy. All subjects in this study were operated on by the same surgeon. Rats received intraperitoneal postoperative antibiotic injections of gentamicin (Covetrus, Portland, ME; 5.0 mg/kg) and 10 mL of lactated Ringer solution (Baxter, Deerfield, IL) to prevent dehydration. Antibiotic injections were given once daily on the day of surgery and for 6 days afterward. Lactated Ringer solution was administered twice daily on the day of surgery and for 2 days afterward. Postoperative analgesics were not given to avoid confounding results of pain outcomes. For 1 week after surgery, rats were placed on a water-jacketed heating pad at 38°C and bladders were expressed manually twice daily until recovery of bladder function was observed.

### 2.3. von Frey and tail flick testing

For von Frey testing, rats were acclimatized to a plexiglass chamber with a wire mesh floor for 30 min/session for 5 days before collecting baseline data. After SCI, von Frey testing was done at 5 weeks to ensure all rats were able to bear weight on their hind paws. To induce a withdrawal response, a series of von Frey filaments (4–100 g) (Stoelting, Wood Dale, IL) were placed on the plantar surface between the foot pads of each hind paw for 1 second, allowing 30 seconds between each stimulus, resulting in 10 trials per session on testing days. Before application, the tester ensured all 4 paws were on the mesh floor to ensure even distribution of weight. The score from each filament depends upon a positive or negative response, known as the up–down method as previously described.^21^ The 50% withdrawal threshold was calculated at baseline and after each session from weeks 5 to 8. Trials in which the stimulus appeared to lift the paw up off the grid or was applied to the foot pad were excluded and repeated. This test was performed by the same 2 investigators throughout the experiment to control for interrater variability.

For the tail flick test, rats were handled daily 1 week before testing for 5 minutes a day. A water bath (Fisher Scientific, Pittsburgh, PA) was maintained at 50°C and each rat's tail was placed in the water bath while the rat was held in the tester's hand in a relaxed state.^30^ The tail flick latency was measured, defined as the period between when the rat's tail was submerged, and a withdrawal response was observed. The tail flick test was performed at baseline and at 1 day postinjury (dpi) and weekly thereafter from 1 to 8 weeks post injury (wpi). Each rat's tail was held for no longer than 10 seconds without a withdrawal in the water bath to avoid injury.

### 2.4. CatWalk testing

Gait analysis was measured with the CatWalk XT system (Noldus, Leesburg, VA) at baseline and weekly from weeks 5 to 8 postinjury once the rats were able to perform consistent hindlimb stepping. Rats underwent training on the CatWalk platform for 5 days or until they could cross the platform with consistent stepping, as previously described.^17^ During acquisition at baseline and after injury, 3 compliant runs were collected for each rat. An automated analysis of the gait was performed by the software, followed by manual corrections seen by the investigator. Run duration (s), regularity index (%), base of support (cm), and print area (cm^2^) were calculated after acquisitions were complete. Run duration was measured as the number of seconds required for the rat to cross the distance of the platform. Regularity index was measured as a percentage of the number of normal step sequence patterns divided by the number of paw placements. Base of support was measured in cm as the distance between the hind paws. Print area was measured as the complete paw print area of the hind paws while walking in cm^2^.

### 2.5. Flow cytometry

Rats (n = 6 sham, n = 16 SCI) were randomly assigned into groups for flow cytometry and immunohistochemistry, described below. For flow cytometry, rats were injected with 0.3 mL of sodium pentobarbital solution (150 mg/kg; Covetrus). Once anesthetized, rats were decapitated, and 10.0 mm of spinal cord tissue centered at the injury epicenter (T9) was dissected and processed as previously described.^19^ The tissue was placed into MACS Tissue Storage Solution (Miltenyi Biotec, Bergisch Gladbach, Germany) at 4°C, and samples were dissociated using the gentleMACS Octo Dissociator and enzymatic solution for 45 minutes at 37°C. Dissociated samples were filtered through 70-µm cell strainers and purified by adding Debris Removal Solution (Miltenyi Biotec), followed by Red Blood Cell Lysis Solution (Miltenyi Biotec). Cells underwent live/dead staining (Invitrogen, Waltham, MA; L434966A) before being labeled with conjugated antibodies, after which they were resuspended in 2% paraformaldehyde solution and stored overnight at 4°C. The following antibodies were used in the flow cytometry panel: BV412-CD11b/c (BD Biosciences, Franklin Lakes, NJ; 743977), BB700-CD45 (BD Biosciences; 742159), BV605-CD80 (BD Biosciences; 743865), BV786-CD86 (BD Biosciences; 743216), FITC-CD3 (BD Biosciences; 554832), PE-CD68 (Invitrogen; 16653), Biotin-CD163 (Novus, Minneapolis, MN; NBP2-39099B), and APC-Streptavidin (BD Biosciences; 554067).

Samples were run on an LSR Fortessa flow cytometer (BD Biosciences). One million events were collected per sample. Acquisition was monitored using the FACS Diva software (BD Biosciences) and data analysis was performed using FlowJo software (BD Biosciences). We isolated CD11b^+^/CD45^low^ as microglia and CD11b^+^/CD45^high^ population as infiltrating macrophages, as previously described.^29,40^ It is important to note that, given the ability of microglia to modulate CD45 expression based on their activation state, the “macrophage” (CD11b^+^, CD45^high^) population defined by our gating strategy likely also includes activated microglia. The “microglia” (CD11b^+^, CD45^low^) population, rather, likely includes only the nonactivated, ramified microglia. Displaying myeloid events on the CD45 vs CD11b view alone, the distinction between the CD45^low^ and CD45^high^ groups is not apparent (Supplemental Figure 1, http://links.lww.com/PR9/A248). However, if the myeloid events are displayed on the CD45 vs forward scatter (FSC) view, the CD45^low^ events exhibit lower FSC than the CD45^high^ events, allowing for the 2 populations to be delineated (Supplemental Figure 1, http://links.lww.com/PR9/A248). The position of the delineation was also informed by the intensity of the CD45 channel observed in fluorescence minus one (FMO) control data.

### 2.6. Immunohistochemistry

Once anesthetized as described above, rats (n = 5 sham, n = 8 SCI) were transcardially perfused with 4% paraformaldehyde as previously described.^20^ A 2.0-cm length of the spinal cord centered at the injury epicenter (T9) was cryoprotected in 30% sucrose, embedded in OCT embedding matrix, sectioned on a cryostat at 20-µm thickness, and thaw-mounted onto electrostatically charged slides.

For immunofluorescence staining, slides were washed 3 times for 5 minutes with phosphate-buffered saline (PBS) with 0.3% Triton then blocked with 10% normal goat serum and 0.3% Triton for 2 hours. The slides were then incubated overnight in primary antibody CD68 (1:500; MA5-16654; Invitrogen). The next day, slides were washed with PBS before incubation with secondary antibody Alexa Flour 568 anti-mouse (1:100; A11004; Invitrogen) for 2 hours at room temperature. Tissue was washed again in PBS before applying the green FlouroMyelin stain (1:30; F34651; Invitrogen) for 20 minutes at room temperature. Tissue was washed with PBS once more before counterstaining with DAPI and cover slipping (ProLong Gold).

Every 20th tissue section throughout the entire lesion (defined as the number of sections where the lesion was present) was quantified for both the number of CD68^+^ cells (Optical Fractionator) and the amount of spared white matter (Cavalieri Estimator) using StereoInvestigator (MBF Bioscience, Williston, VT) on a fluorescent microscope (Olympus, Tokyo, Japan). Spared white matter was defined as tissue that had green FlouroMyelin staining. CD68^+^ cells were defined as red fluorescent cells with nuclei counterstained with DAPI (200 µm × 200 µm grid size; 50 µm × µm sampling box size). Injured tissue prompts infiltrating macrophages to undergo a morphological transformation, adopting an amoeboid form.^29^ Representative images of tissue sections for figures were acquired using StereoInvestigator. The same settings were used to acquire all images.

### 2.7. Data analysis

All data are shown as the mean ± SEM. Analysis was performed using GraphPad Prism v10 and IBM SPSS v26 software. Comparisons between groups were performed using a one-way analysis of variance (ANOVA) followed by a Tukey *post hoc* test, a 2-way ANOVA followed by a Sidak *post hoc* test, or an unpaired Student *t*-test. For the hierarchical cluster analysis, the distance between groups was assessed using the squared Euclidean distance to classify normosensitive rats or hypersensitive/hyposensitive rats. We followed up with a K-means cluster analysis, as seen in Supplemental Tables 1 and 2, http://links.lww.com/PR9/A249 to show which of the timepoints in each subgroup was significantly different in the hypersensitive/hyposensitive rats. *P*-values less than 0.05 were considered statistically significant).

## 3. Results

### 3.1. Defining thermal and mechanical sensitive rats after spinal cord injury

**Figure 1.**
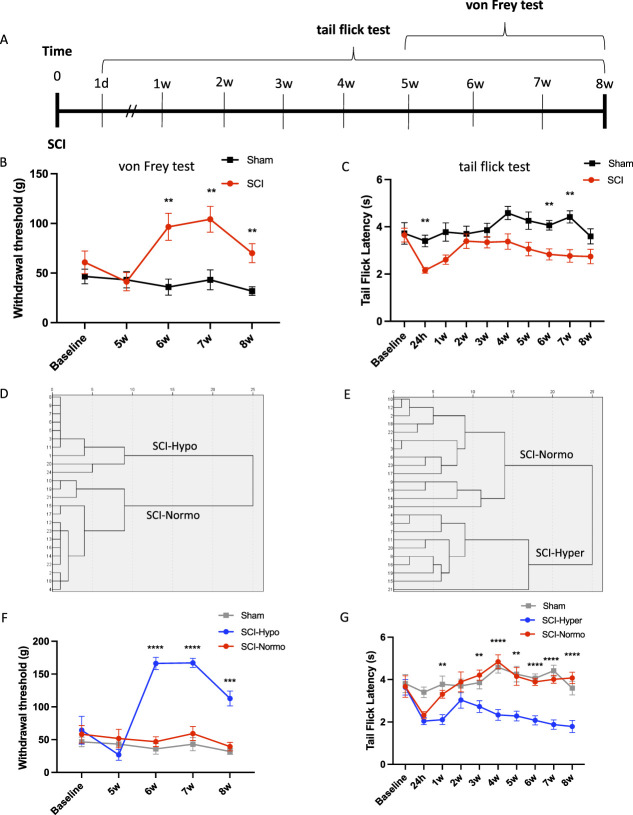
Identifying rats with sensitivity to thermal and mechanical stimuli after spinal cord injury (SCI). (A) Schematic diagram showing the testing schedule after baseline behavior was recorded and SCI was performed on day 0. The tail flick test was performed at 1 day (d) postinjury and weekly until 8 weeks (w). The von Frey test was performed weekly from weeks 5 to 8. (B) SCI rats (n = 27) tested for sensitivity to mechanical stimuli using the von Frey test show a significantly higher withdrawal threshold starting at 6 weeks after injury until 8 weeks compared with sham rats (n = 11). **P < 0.005 by Sidak *post hoc* test, following 2-way ANOVA. (C) Rats tested for thermal sensitivity to mechanical stimuli show a significant decrease in tail flick latency at 24 hours, 6 weeks, and 7 weeks. **P < 0.005 by Sidak *post hoc* test, following 2-way ANOVA. (D) Dendrogram of hierarchical cluster analysis revealed 2 distinct groups of SCI rats determined to have hyposensitivity and normosensitivity to mechanical stimuli. (E) A dendrogram of hierarchical cluster analysis reveals 2 distinct groups of SCI rats determined to have hypersensitivity and normosensitivity to thermal stimuli. (F) When replotting these subgroups, rats with high withdrawal thresholds (SCI-Hypo) are significantly different than the sham rats starting at 6 weeks. ***P < 0.0005 by Sidak *post hoc* test, following 2-way ANOVA. (G) Similarly, rats showing decreased tail flick latency (SCI-Hyper) are significantly different from the sham rats, whereas the SCI-normosensitive rats are not. **P < 0.005 by Sidak *post hoc* test, following 2-way ANOVA.

A total of 24 rats that received a spinal cord injury were tested for both sensitivity to mechanical stimuli, using the up-down von Frey test, and sensitivity to thermal stimuli, using the tail flick test, up to 8 weeks postinjury (Fig. 1A). Spinal cord injury rats showed significantly greater withdrawal thresholds from weeks 6 to 8 (Fig. 1B) and significantly decreased tail flick latency at 24 hours, 6 and 7 weeks after injury (Fig. 1C) indicating sensory abnormalities after injury. To further evaluate differences in sensory abnormalities, we used a hierarchal cluster analysis to identify any subgroups of the SCI population (Fig. 1D, E). There were 2 significantly different subgroups for withdrawal threshold outcomes. The analysis showed 10 rats with decreased sensitivity to mechanical stimuli, presenting with a higher withdrawal threshold compared with sham rats from weeks 7 to 8 (*P* < 0.0005). These rats were labeled as SCI-Hypo because they showed a hyposensitive response after injury. Then, 14 rats showed similar withdrawal threshold scores to sham rats and were labeled as SCI-Normo because of their normosensitive response (Fig. 1F). Similarly, 2 populations of rats were identified for tail-flick latency scores. There were 14 rats that showed an increased sensitivity to the thermal stimuli compared with sham rats on week 1 and weeks 3 to 8 (labeled as SCI-Hyper because of their hypersensitive response). The other 10 rats, however, showed similar tail flick latency scores to sham rats and were labeled as SCI-Normo because of their normosensitive response (*P* < 0.005; Fig. 1G).

### 3.2. Inflammatory cells at the injury epicenter in mechanical and thermal sensory phenotypes

**Figure 2.**
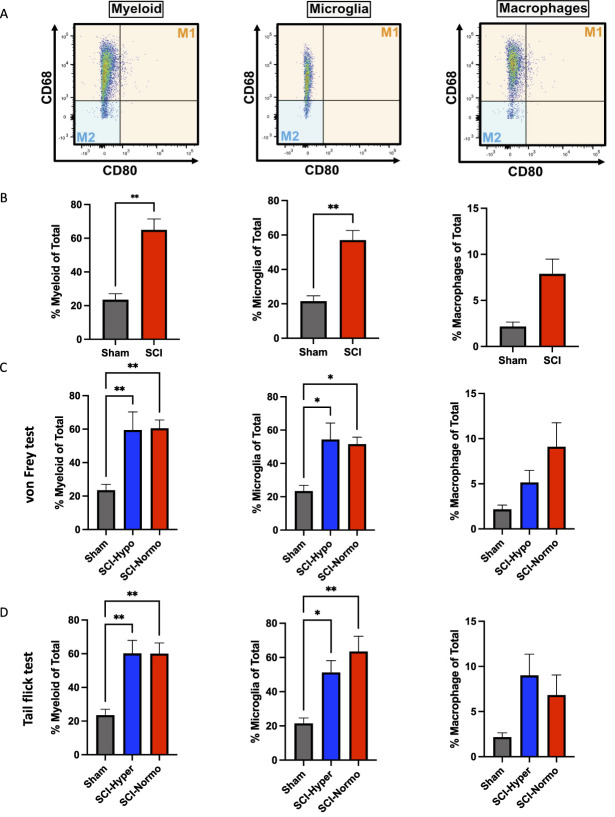
Inflammation in rats with mechanical and thermal sensory phenotypes. (A) Flow cytometry gating strategy used to identify myeloid, microglia, and macrophage population through expression of CD68 and CD80. (B) SCI rats (n = 16) have a significantly increased percent myeloid and microglia compared with sham rats (n = 6) **P < 0.005 by Student *t* test. There is no significant difference in the percent macrophages in SCI rats compared with sham. (C) There are no significant differences in inflammatory markers between rats with hyposensitivity and rats with normosensitivity to mechanical stimuli. Both the SCI subgroups of mechanical sensitivity have significantly increased percent myeloid and microglia compared with sham. *P < 0.05 by Tukey *post hoc*, following 1-way ANOVA. (D) There are no significant differences in inflammatory markers between rats with hypersensitivity and rats with normosensitivity to thermal stimuli. Both the SCI subgroups of thermal sensitivity have significantly increased percent myeloid and percent microglia compared with sham. *P < 0.05 by Tukey post hoc, following 1-way ANOVA. SCI, spinal cord injury.

To assess if inflammatory cells affected the development of CNP 8 weeks after SCI, we used flow cytometry to measure the percentage of the myeloid, microglia, and macrophage cell populations at the injury epicenter (Fig. 2A). There is a significant increase in the percentage of myeloid cells and microglia (of total cells) when comparing sham rats and SCI rats (*P* < 0.005; Fig. 2B). When assessing differences between experimental groups, a 1-way ANOVA demonstrated a main effect (F[2,19] = 7.993, *P* < 0.005). However, a subsequent post hoc analysis did not show a significant difference between rats with sensitivity to mechanical or thermal stimuli (Fig. 2C, D). There was no significant difference between the percentage of macrophages when comparing SCI and sham rats, likely because of the significant variance within the groups (Fig. 2B). Similarly, we did not see any significant differences when assessing subgroups of mechanical or thermal sensitivity (Fig. 2 C, D).

### 3.3. CD68-positive cells in mechanical and thermal sensory phenotypes

**Figure 3.**
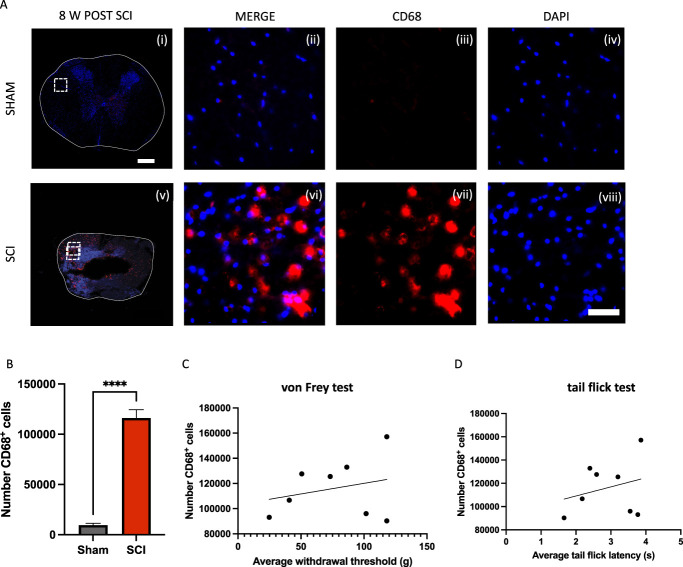
CD68^+^ cells in rats with mechanical and thermal sensory phenotypes. (A) Representative transverse images near the injury epicenter (T9) of CD68^+^ (red) immunofluorescent staining in a sham rat (n = 5) (i–iv) and SCI rat (n = 8) (v–viii) 8 weeks after injury. The nuclei were counterstained with DAPI (blue). (B) There is a significantly greater number of CD68^+^ cells at the injury epicenter in SCI rats compared with sham rats. ***P < 0.0005 by Student *t* test. (C and D) There was no correlation found between the amount of CD68^+^ cells in the spinal cord at the injury epicenter (*r* = 0.2546) and the average withdrawal threshold or average tail-flick latency (*r* = 0.2695). Scale bar represents 400 µm (i) and 50 µm (viii). SCI, spinal cord injury.

Macrophage expression at the injury epicenter was further assessed by CD68 immunofluorescent staining to delineate the presence of M1 macrophages from the variability in percentage of macrophages detected in the flow cytometric analysis (Fig. 3A). There was a significant increase in CD68-positive cells at the injury epicenter in rats after SCI compared with sham rats (*P* < 0.00005; Fig. 3B). But there was no correlation seen in rats with sensitivity to either mechanical or thermal stimuli.

### 3.4. White matter sparing in mechanical and thermal sensory phenotypes

**Figure 4.**
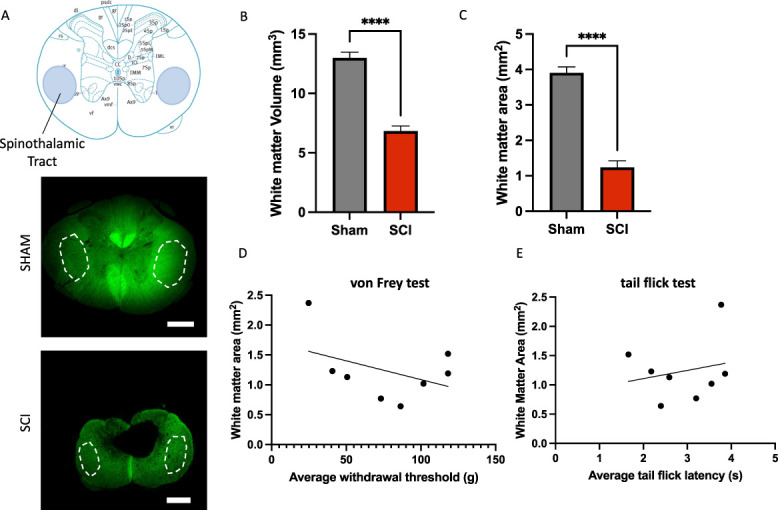
White matter volume in rats with mechanical and thermal sensory phenotypes. (A) Representative histological sections of FlouroMyelin (green) from a sham rat (n = 5) and SCI rat (n = 8) near the injury epicenter 8 weeks postinjury. The outline delineates the spinothalamic and corticospinal tracts displayed on the atlas of the rat spinal cord at T9 on the top image. (B) SCI rats have significantly less white matter volume compared with sham rats. ****P < 0.00005 by Student *t* test. (C) SCI rats also have significantly less white matter area in the spinal thalamic tract region compared to sham rats. ****P < 0.00005 by Student *t* test. (D and E) There was no correlation found between the amount of spared white matter in the spinal cord at the injury epicenter and the average withdrawal threshold (*r* = −0.4183) or average tail flick latency (*r* = 0.2130). Scale bar represents 400 µm. SCI, spinal cord injury.

Next, we measured white matter area to assess whether the degree of injury had any impact on the development of sensory abnormalities after SCI. The white matter volume was measured using quantification of fluorescent myelin at the injury epicenter (Fig. 4A). There was a significant decrease in the volume of white matter at the injury epicenter when comparing SCI rats to sham rats (*P* < 0.00005; Fig. 4B). We also assessed white matter sparing in the lateral funiculi, to capture the spinothalamic tract, because degeneration of this area is known to alter transmission of sensory information and promote central sensitization, respectively, contributing to neuropathic pain.^24,32,45^ However, there was no correlation between the degree of white matter sparing and sensitivity to mechanical or thermal stimuli (Fig. 4C). This indicates that the number of spared myelinated axons in the lateral spinal cord white matter alone does not affect the development of either CNP phenotype.

### 3.5. Central neuropathic pain phenotypes and locomotor recovery after spinal cord injury

**Figure 5.**
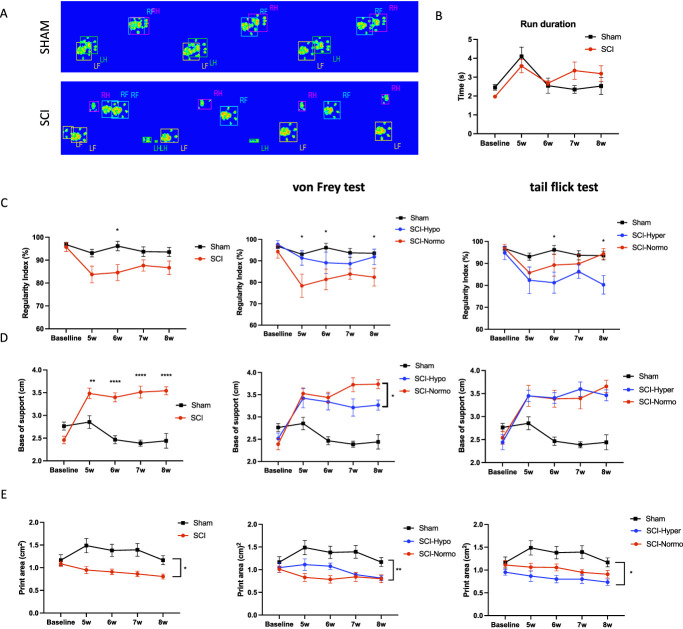
Locomotor recovery in SCI sensory phenotypes. (A) Graphical print view of CatWalk XT automated gait analysis system in sham rat and SCI rat. (B) Run duration is not significantly different between sham rats and SCI rats. (C) Regularity index is significantly decreased at 6 weeks post injury in SCI rats compared with sham rats. When analyzing subgroups of rats SCI rats, those with normosensitivity to mechanical stimuli show a significant difference from sham rats at 5, 6, and 8 weeks (*P < 0.05), whereas the hyposensitive rats do not. Rats with hypersensitivity to mechanical stimuli show a significantly decreased regularity index compared with sham rats at 6 and 8 weeks (*P < 0.05) compared with the normosensitive rats that do not show any differences. (D) SCI rat base of support is significantly different from sham rats (**P < 0.005). Rats with hyposensitivity to mechanical stimuli show a significant decrease in base of support compared with normosensitive rats at 7 and 8 weeks (*P < 0.05). There are no significant differences in rats with hypersensitivity vs normosensitivity to thermal stimuli in base of support. (E) SCI rat print area is significantly different from sham rats (*P < 0.05). Rats with normosensitivity to mechanical stimuli have a significant decrease in print area at 5 and 6 weeks compared with sham rats (*P < 0.05). Rats with hypersensitivity to thermal stimuli have a significant decrease in print area from 5 to 8 weeks compared with sham rats (*P* < 0.05). Significance by Sidak post hoc after 2-way ANOVA (C–E). SCI, spinal cord injury.

Finally, we wanted to assess the impact of CNP phenotypes on locomotor recovery using the CatWalk test to assess multiple parameters of gait (Fig. 5A). The first parameter we investigated was run duration (the time it takes a given rat to cross the platform). When comparing sham vs SCI rats (Fig. 5B), there was no significant difference at any timepoint, showing that the degree of injury in this study does not affect the walking speed and therefore should not be a confounding factor for the additional gait parameters that were assessed.

We see that there is only a significant decrease in regularity index on week 6 when comparing sham with SCI rats (*P* < 0.05; Fig. 5C). However, a breakdown of the subgroups for hyposensitive and normosensitive rats to mechanical stimuli using the von Frey test show that normosensitive rats have a significant decrease in regularity index on weeks 5, 6, and 8 compared with sham rats (*P* < 0.05). These results suggest that the normosensitive phenotype, or rats that remain sensitive to mechanical stimuli similarly to sham rats, is disadvantageous to motor recovery compared with rats that had a significant reduction in sensation to mechanical stimuli. Conversely, we see that the rats that are normosensitive to thermal stimuli do not show any significant differences in regularity index compared with sham rats, but it is the hypersensitive rats that show a significant decrease in regularity index on weeks 6 and 7 (*P* < 0.05).

Hind limb base of support increases after spinal cord injury across weeks 5 through 8 (Fig. 5D). Consistent with the findings from regularity index, we found that rats that are normosensitive to mechanical stimuli had a more significant increase in base of support on weeks 7 and 8 compared with sham rats than was demonstrated by the hyposensitive rats. There were no significant differences between subgroups of thermal sensitive rats compared with sham rats at any timepoints.

Print area is decreased from weeks 5 to 8 when comparing SCI with sham rats as expected because of the impairment of weight supported stepping after injury (*P* < 0.05; Fig. 5E). We see that print area is further impaired in those rats that are normosensitive to mechanical stimuli on weeks 5 to 8 compared with sham rats but only significantly decreased for the hyposensitive rats on weeks 7 and 8 (*P* < 0.05). The results also show that rats that are hypersensitive to thermal stimuli have a significant decrease in print area compared with sham rats (*P* < 0.05), whereas the rats that are normosensitive to thermal stimuli are not significantly different from the sham rats.

## 4. Discussion

Our study revealed that a subset of rats exhibited chronic alterations in sensitivity to mechanical or thermal stimuli up to 8 weeks post SCI. By contrast to the common findings of hypersensitivity in rodents, our study identified a subset of rats with hyposensitivity or sensory loss. Notably, this hyposensitive phenotype did not indicate pain-like behavior, and our results suggested that normosensitive rats to mechanical stimuli exhibited impaired locomotor recovery. In addition, rats showed hypersensitivity to thermal stimuli regardless of their sensitivity to mechanical stimuli. Individual mediated responses highlight the importance of understanding mechanistic influences to develop more effective therapies.

The identification of a subset of rats displaying mechanical hyposensitivity, a phenomenon not commonly reported, challenges existing assumptions and provides a nuanced perspective on sensory outcomes. Several hypotheses were explored to elucidate this phenomenon, including hyperreflexia, injury timing, genetics, and gender. Previous findings indicate that hyperreflexia, linked with hyposensitivity, may signify spasms rather than pain.^26^ In addition, estrogen's role in promoting nerve growth and regeneration suggests its potential influence on pain responses through the estrous cycle.^8^ Genetics play a significant role in pain perception, even within inbred rodent strains.^23,36^ Another study demonstrated that downregulation of Per1, a circadian protein, improved hypersensitivity, suggesting that the timing of SCI could affect pain outcomes.^37^ Moreover, physiological factors such as individual stress, anxiety, and depression may also influence pain perception, emphasizing the need for further investigation into these aspects.^4,27,42,43^

It is essential to note the limitations of our study, particularly regarding the von Frey test methodology. Variability in testing methodologies across the field, including differences in gender, strain, weight, and contusion force/dwell time can all influence outcomes.^13,31,35^ Although this study used the application of a 1-second von Frey filament stimulation, as described by Detloff et al., other studies have used a 5-second stimulation.^21,39^ Furthermore, we used a 200-kdyn (0 seconds dwell time) contusion to interrogate whether there was a clear pain effect on locomotor recovery. Although Carter et al. demonstrated that both 150- and 200-kdyn contusions in rats result in mechanical hypersensitivity, they showed a greater hypersensitive change from baseline in the 150 kdyn that had a dwell time of at least 1 second.^13^ In addition, the exponential jump in forces between monofilaments and changes in filament diameter could affect sensory behavior.

In cellular responses, a significant increase in myeloid and microglia cell populations were observed after SCI. These changes, however, lacked the specificity needed to differentiate the onset of mechanical sensitivity vs thermal sensitivity in rats. We did see a significant degree of variability in macrophage expression at the injury epicenter, but histological analysis of CD68^+^ cells could not be used to indicate sensory phenotypes. Quantification of white matter area at the injury site did not correlate to the degree of mechanical or thermal sensitivity, indicating that damage to the spinothalamic tract alone is likely not sufficient for the development of CNP sensory phenotypes. This is consistent with several other studies in rodents, and humans, which show damage to spinothalamic and corticospinal tracts are necessary for the development of CNP but do not correlate to the degree of pain.^15,32,45^ Although we chose to focus on the spinal mechanisms of inflammation, future studies need to consider the peripheral mechanisms of SCI-induced neuropathic pain phenotypes as previous studies have shown inflammatory and transcriptional changes to dorsal root ganglia, and hyperactivity of primary nociceptors, may play a role in promoting central neuropathic pain.^12,16,33,48^ We also recognize the limitations of our study in directly linking site-specific immune responses to behavioral changes in the lumbar and sacral regions despite acknowledging the systemic response of immune cells after SCI and the role of neural plasticity in response to SCI.^1,38,47^

The CatWalk analysis allowed us to determine the impact of locomotor deficits from rats with different sensory phenotypes without having to control for differences in speed because it has been shown that walking speed can affect gait patterns.^7^ It appeared that rats that were normosensitive to mechanical stimuli and hypersensitive to thermal stimuli had more deficits in locomotor parameters compared with their counterparts. Partly, this can be attributed to an increase in inflammation in CNP phenotypes, not only affecting central sensitization in nociceptive pathways but also those pathways related to motor learning.^34^

In the context of SCI, managing secondary symptoms like pain is crucial for enhancing quality of life. However, current pharmacotherapies and surgeries for CNP after SCI have shown limited efficacy.^11,14^ Most studies have been unsatisfactory in providing clinically translatable animal models by using unrealistic injury types or relying solely on alterations in mechanically evoked responses as a measure of pain.^6,9^ Our study used a clinically relevant contusion model and used 2 sensory tests, elaborating on distinctive hyposensitive findings to catalyze further research.

In summary, this study assessed histopathological and locomotor outcomes of 2 sensory phenotypes for mechanical and thermal tests after SCI. Our findings show that inflammatory cells at the site of the injury do not affect sensory phenotypes in female rats after a contusive SCI. We also show that pain phenotypes impair locomotor outcomes to varying degrees depending on the gait parameter when comparing each phenotype to sham rats. Our data highlight the importance of distinguishing biomarkers for individual pain phenotypes and understanding their relationship to recovery to translate these findings to humans and develop the most effective treatment strategies.

## Disclosures

The authors have no conflict of interest to declare.

## Appendix A. Supplemental digital content

Supplemental digital content associated with this article can be found online at http://links.lww.com/PR9/A248 and http://links.lww.com/PR9/A249.

## References

[1] Andrade MSR Hanania FR Daci K Leme RJA Chadi G. Contuse lesion of the rat spinal cord of moderate intensity leads to a higher time-dependent secondary neurodegeneration than severe one. An open-window for experimental neuroprotective interventions. Tissue Cell 2008;40:143–56. 18207478 10.1016/j.tice.2007.11.002

[2] Andresen SR Biering-Sørensen F Hagen EM Nielsen JF Bach FW Finnerup NB. Pain, spasticity and quality of life in individuals with traumatic spinal cord injury in Denmark. Spinal Cord 2016;54:973–9. 27067654 10.1038/sc.2016.46

[3] Attal N Mazaltarine G Perrouin-Verbe B Albert T, SOFMER French Society for Physical Medicine and Rehabilitation. Chronic neuropathic pain management in spinal cord injury patients. What is the efficacy of pharmacological treatments with a general mode of administration? (oral, transdermal, intravenous). Ann Phys Rehabil Med 2009;52:124–41. 19909703 10.1016/j.rehab.2008.12.011

[4] Baron R Maier C Attal N Binder A Bouhassira D Cruccu G Finnerup NB Haanpää M Hansson P Hüllemann P Jensen TS Freynhagen R Kennedy JD Magerl W Mainka T Reimer M Rice ASC Segerdahl M Serra J Sindrup S Sommer C Tölle T Vollert J Treede R-D, German Neuropathic Pain Research Network DFNS, and the EUROPAIN, and NEUROPAIN consortia. Peripheral neuropathic pain: a mechanism-related organizing principle based on sensory profiles. PAIN 2017;158:261–72. 27893485 10.1097/j.pain.0000000000000753 PMC5266425

[5] Basso DM Beattie MS Bresnahan JC. A sensitive and reliable locomotor rating scale for open field testing in rats. J Neurotrauma 1995;12:1–21. 7783230 10.1089/neu.1995.12.1

[6] Batista CM Mariano ED Onuchic F Dale CS dos Santos GB Cristante AF Otoch JP Teixeira MJ Morgalla M Lepski G. Characterization of traumatic spinal cord injury model in relation to neuropathic pain in the rat. Somatosensory Mot Res 2019;36:14–23. 10.1080/08990220.2018.1563537 30870070

[7] Batka RJ Brown TJ Mcmillan KP Meadows RM Jones KJ Haulcomb MM. The need for speed in rodent locomotion analyses. Anat Rec (Hoboken) 2014;297:1839–64. 24890845 10.1002/ar.22955 PMC4758221

[8] Blacklock AD Johnson MS Krizsan-Agbas D Smith PG. Estrogen increases sensory nociceptor neuritogenesis in vitro by a direct, nerve growth factor-independent mechanism. Eur J Neurosci 2005;21:2320–8. 15932591 10.1111/j.1460-9568.2005.04075.x

[9] Blumenthal GH Nandakumar B Schnider AK Detloff MR Ricard J Bethea JR Moxon KA. Modelling at‐level allodynia after mid‐thoracic contusion in the rat. Eur J Pain 2021;25:801–16. 33296535 10.1002/ejp.1711 PMC8318779

[10] Burke D Fullen BM Stokes D Lennon O. Neuropathic pain prevalence following spinal cord injury: a systematic review and meta-analysis. Eur J Pain 2017;21:29–44. 27341614 10.1002/ejp.905

[11] Cardenas DD Warms CA Turner JA Marshall H Brooke MM Loeser JD. Efficacy of amitriptyline for relief of pain in spinal cord injury: results of a randomized controlled trial. PAIN 2002;96:365–73. 11973011 10.1016/S0304-3959(01)00483-3

[12] Carlton SM Du J Tan HY Nesic O Hargett GL Bopp AC Yamani A Lin Q Willis WD Hulsebosch CE. Peripheral and central sensitization in remote spinal cord regions contribute to central neuropathic pain after spinal cord injury. PAIN 2009;147:265–76. 19853381 10.1016/j.pain.2009.09.030 PMC2787843

[13] Carter MW Johnson KM Lee JY Hulsebosch CE Gwak YS. Comparison of mechanical allodynia and recovery of locomotion and bladder function by different parameters of low thoracic spinal contusion injury in rats. Korean J Pain 2016;29:86–95. 27103963 10.3344/kjp.2016.29.2.86 PMC4837124

[14] Cofano F Boido M Monticelli M Zenga F Ducati A Vercelli A Garbossa D. Mesenchymal stem cells for spinal cord injury: current options, limitations, and future of cell therapy. Int J Mol Sci 2019;20:2698. 31159345 10.3390/ijms20112698 PMC6600381

[15] Cruz-Almeida Y Felix ER Martinez-Arizala A Widerström-Noga EG. Decreased spinothalamic and dorsal column medial lemniscus-mediated function is associated with neuropathic pain after spinal cord injury. J Neurotrauma 2012;29:2706–15. 22845918 10.1089/neu.2012.2343 PMC3510448

[16] Cuevas-Diaz Duran R Li Y Garza Carbajal A You Y Dessauer CW Wu J Walters ET. Major differences in transcriptional alterations in dorsal root ganglia between spinal cord injury and peripheral neuropathic pain models. J Neurotrauma 2023;40:883–900. 36178348 10.1089/neu.2022.0238 PMC10150729

[17] David BT Curtin JJ Brown JL Scorpio K Kandaswamy V Coutts DJC Vivinetto A Bianchimano P Karuppagounder SS Metcalfe M Cave JW Hill CE. Temporary induction of hypoxic adaptations by preconditioning fails to enhance Schwann cell transplant survival after spinal cord injury. Glia 2023;71:648–66. 36565279 10.1002/glia.24302 PMC11848738

[18] David BT Curtin JJ Goldberg DC Scorpio K Kandaswamy V Hill CE. Hypoxia-inducible factor 1α (HIF-1α) counteracts the acute death of cells transplanted into the injured spinal cord. eNeuro 2020;7:ENEURO.0092-19.2019. 10.1523/ENEURO.0092-19.2019 PMC7215587 31488552

[19] David BT Ratnayake A Amarante MA Reddy NP Dong W Sampath S Heary RF Elkabes S. A toll-like receptor 9 antagonist reduces pain hypersensitivity and the inflammatory response in spinal cord injury. Neurobiol Dis 2013;54:194–205. 23313320 10.1016/j.nbd.2012.12.012

[20] David BT Steward O. Deficits in bladder function following spinal cord injury vary depending on the level of the injury. Exp Neurol 2010;226:128–35. 20713043 10.1016/j.expneurol.2010.08.014 PMC2955760

[21] Detloff MR Clark LM Hutchinson KJ Kloos AD Fisher LC Basso DM. Validity of acute and chronic tactile sensory testing after spinal cord injury in rats. Exp Neurol 2010;225:366–76. 20643128 10.1016/j.expneurol.2010.07.009 PMC4933012

[22] Deuis JR Dvorakova LS Vetter I. Methods used to evaluate pain behaviors in rodents. Front Mol Neurosci 2017;10:284. 28932184 10.3389/fnmol.2017.00284 PMC5592204

[23] Devor M. How does gabapentin relieve neuropathic pain? PAIN 2009;145:259–61. 19539428 10.1016/j.pain.2009.05.027

[24] Finnerup N Pedersen L Terkelsen A Johannesen I Jensen T. Reaction to topical capsaicin in spinal cord injury patients with and without central pain. Exp Neurol 2007;205:190–200. 17346705 10.1016/j.expneurol.2007.01.026

[25] Gaudet AD Ayala MT Schleicher WE Smith EJ Bateman EM Maier SF Watkins LR. Exploring acute-to-chronic neuropathic pain in rats after contusion spinal cord injury. Exp Neurol 2017;295:46–54. 28552717 10.1016/j.expneurol.2017.05.011

[26] van Gorp S Deumens R Leerink M Nguyen S Joosten EA Marsala M. Translation of the rat thoracic contusion model; part 1-supraspinally versus spinally mediated pain-like responses and spasticity. Spinal Cord 2014;52:524–8. 24819511 10.1038/sc.2014.72

[27] Gruener H Zeilig G Laufer Y Blumen N Defrin R. Increased psychological distress among individuals with spinal cord injury is associated with central neuropathic pain rather than the injury characteristics. Spinal Cord 2018;56:176–84. 29238095 10.1038/s41393-017-0014-6

[28] Khan MI Arsh A Ali I Afridi AK. Frequency of neuropathic pain and its effects on rehabilitation outcomes, balance function and quality of life among people with traumatic spinal cord injury. Pak J Med Sci 2022;38:888–92. 35634589 10.12669/pjms.38.4.4681 PMC9121970

[29] Kobayakawa K Ohkawa Y Yoshizaki S Tamaru T Saito T Kijima K Yokota K Hara M Kubota K Matsumoto Y Harimaya K Ozato K Masuda T Tsuda M Tamura T Inoue K Edgerton VR Iwamoto Y Nakashima Y Okada S. Macrophage centripetal migration drives spontaneous healing process after spinal cord injury. Sci Adv 2019;5:eaav5086. 31106270 10.1126/sciadv.aav5086 PMC6520026

[30] Kopruszinski CM Swiokla J Lee YS Navratilova E VanderVeen L Yang M Liu Y Miyazaki T Schmidt WK Zalevsky J Porreca F. Preclinical assessment of the analgesic pharmacology of NKTR-181 in rodents. Cell Mol Neurobiol 2021;41:949–60. 32107752 10.1007/s10571-020-00816-3 PMC11448559

[31] Lee SE Greenough EK Oancea P Scheinfeld AR Douglas AM Gaudet AD. Sex differences in pain: spinal cord injury in female and male mice elicits behaviors related to neuropathic pain. J Neurotrauma 2023;40:833–44. 36719772 10.1089/neu.2022.0482

[32] Liu Y Latremoliere A Li X Zhang Z Chen M Wang X Fang C Zhu J Alexandre C Gao Z Chen B Ding X Zhou J-Y Zhang Y Chen C Wang KH Woolf CJ He Z. Touch and tactile neuropathic pain sensitivity are set by corticospinal projections. Nature 2018;561:547–50. 30209395 10.1038/s41586-018-0515-2 PMC6163083

[33] McKay SM McLachlan EM. Inflammation of rat dorsal root ganglia below a mid-thoracic spinal transection. Neuroreport 2004;15:1783–6. 15257147 10.1097/01.wnr.0000135700.52904.77

[34] Mercier C Roosink M Bouffard J Bouyer LJ. Promoting gait recovery and limiting neuropathic pain after spinal cord injury. Neurorehabil Neural Repair 2017;31:315–22. 27913797 10.1177/1545968316680491 PMC5405804

[35] Mills CD Hains BC Johnson KM Hulsebosch CE. Strain and model differences in behavioral outcomes after spinal cord injury in rat. J Neurotrauma 2001;18:743–56. 11526981 10.1089/089771501316919111

[36] Mogil JS Wilson SG Bon K Lee SE Chung K Raber P Pieper JO Hain HS Belknap JK Hubert L Elmer GI Chung JM Devor M. Heritability of nociception II. “Types” of nociception revealed by genetic correlation analysis. PAIN 1999;80:83–93. 10204720 10.1016/s0304-3959(98)00196-1

[37] Morioka N Saeki M Sugimoto T Higuchi T Zhang FF Nakamura Y Hisaoka-Nakashima K Nakata Y. Downregulation of the spinal dorsal horn clock gene Per1 expression leads to mechanical hypersensitivity via c-jun N-terminal kinase and CCL2 production in mice. Mol Cell Neurosci 2016;72:72–83. 26808220 10.1016/j.mcn.2016.01.007

[38] Nesic O Lee J Johnson KM Ye Z Xu G-Y Unabia GC Wood TG McAdoo DJ Westlund KN Hulsebosch CE Regino Perez-Polo J. Transcriptional profiling of spinal cord injury-induced central neuropathic pain. J Neurochem 2005;95:998–1014. 16219025 10.1111/j.1471-4159.2005.03462.x

[39] Odem MA Lacagnina MJ Katzen SL Li J Spence EA Grace PM Walters ET. Sham surgeries for central and peripheral neural injuries persistently enhance pain-avoidance behavior as revealed by an operant conflict test. PAIN 2019;160:2440–55. 31323014 10.1097/j.pain.0000000000001642 PMC6800634

[40] Saiwai H Ohkawa Y Yamada H Kumamaru H Harada A Okano H Yokomizo T Iwamoto Y Okada S. The LTB4-BLT1 axis mediates neutrophil infiltration and secondary injury in experimental spinal cord injury. Am J Pathol 2010;176:2352–66. 20304963 10.2353/ajpath.2010.090839 PMC2861100

[41] Siddall PJ McClelland JM Rutkowski SB Cousins MJ. A longitudinal study of the prevalence and characteristics of pain in the first 5 years following spinal cord injury. PAIN 2003;103:249–57. 12791431 10.1016/S0304-3959(02)00452-9

[42] Tozaki-Saitoh H Masuda J Kawada R Kojima C Yoneda S Masuda T Inoue K Tsuda M. Transcription factor MafB contributes to the activation of spinal microglia underlying neuropathic pain development. Glia 2019;67:729–40. 30485546 10.1002/glia.23570

[43] Tramullas M Francés R de la Fuente R Velategui S Carcelén M García R Llorca J Hurlé MA. MicroRNA-30c-5p modulates neuropathic pain in rodents. Sci Transl Med 2018;10:eaao6299. 30089634 10.1126/scitranslmed.aao6299

[44] Tsuda M. Microglia in the spinal cord and neuropathic pain. J Diabetes Invest 2016;7:17–26. 10.1111/jdi.12379 PMC4718109 26813032

[45] Wasner G Lee BB Engel S McLachlan E. Residual spinothalamic tract pathways predict development of central pain after spinal cord injury. Brain 2008;131:2387–400. 18669485 10.1093/brain/awn169

[46] Widerström-Noga EG Felipe-Cuervo E Yezierski RP. Chronic pain after spinal injury: interference with sleep and daily activities. Arch Phys Med Rehabil 2001;82:1571–7. 11689978 10.1053/apmr.2001.26068

[47] Wu D Miyamoto O Shibuya S Mori S Norimatsu H Janjua NA Itano T. Co-expression of radial glial marker in macrophages/microglia in rat spinal cord contusion injury model. Brain Res 2005;1051:183–8. 15993386 10.1016/j.brainres.2005.05.054

[48] Yang Q Wu Z Hadden JK Odem MA Zuo Y Crook RJ Frost JA Walters ET. Persistent pain after spinal cord injury is maintained by primary afferent activity. J Neurosci 2014;34:10765–9. 25100607 10.1523/JNEUROSCI.5316-13.2014 PMC4122805

