# Supplementary figures and images for: The relationship between changes in inflammation and locomotor function in sensory phenotypes of central neuropathic pain after spinal cord injury
Source: Pain Rep. 2024 Oct 10;9(6):e1184. doi: 10.1097/PR9.0000000000001184 (PMC11469887; doi:10.1097/PR9.0000000000001184)

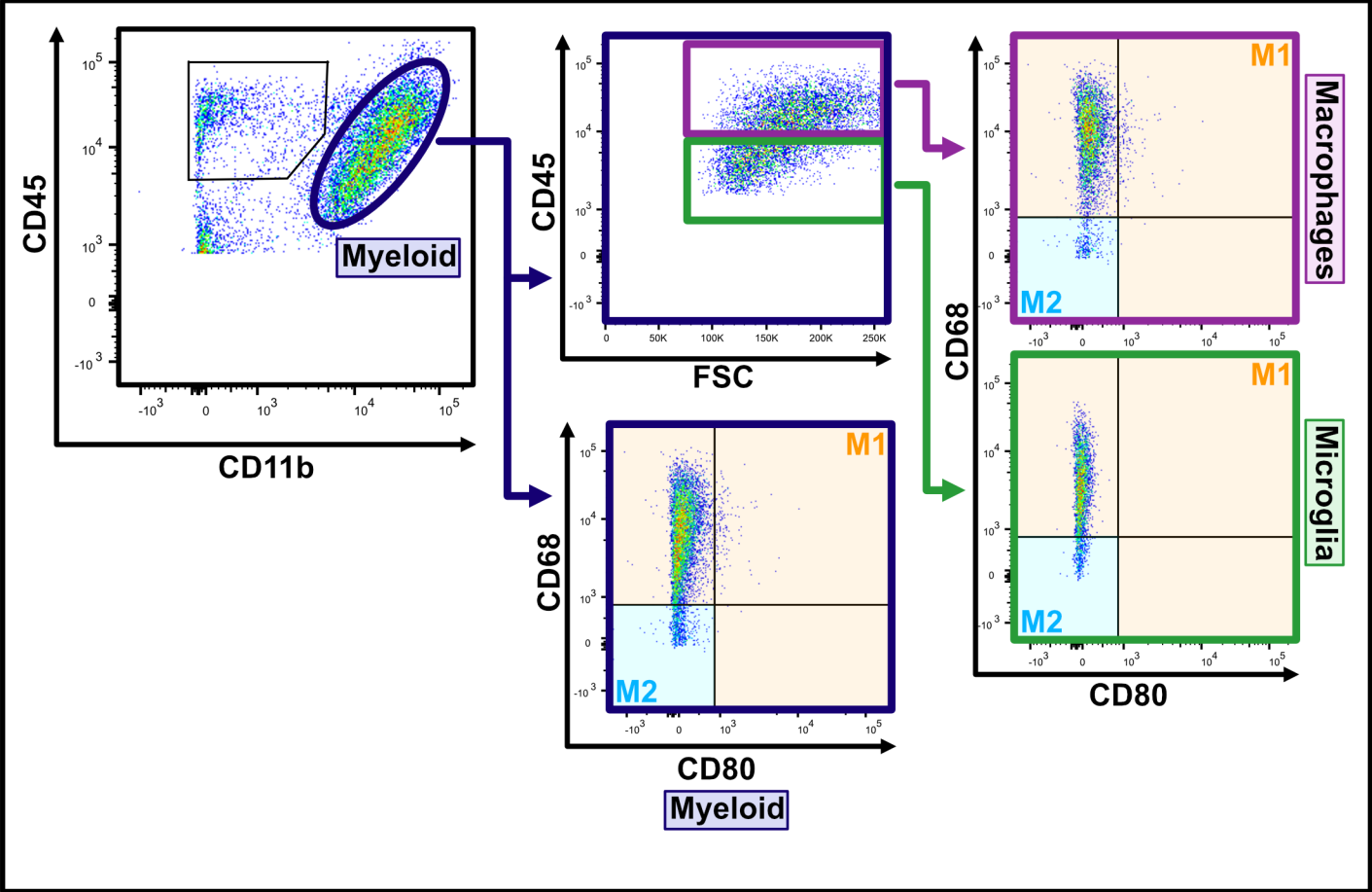

Supplement: Supplementary file 1 [file painreports-9-e1184-s001.pdf]
